# Genome Size of 17 Species From Caelifera (Orthoptera) and Determination of Internal Standards With Very Large Genome Size in Insecta

**DOI:** 10.3389/fphys.2020.567125

**Published:** 2020-10-22

**Authors:** Ying Mao, Nan Zhang, Yimeng Nie, Xue Zhang, Xuejuan Li, Yuan Huang

**Affiliations:** College of Life Sciences, Shaanxi Normal University, Xi’an, China

**Keywords:** Caelifera, evolution, flow cytometry, genome size, internal standard, k-mer analysis

## Abstract

Comparative studies of insect genome size show that Orthoptera is a unique group of Insecta with a significantly enlarged genome. To determine a suitable internal standard for a large genome and to compare the effects of different internal standards on estimates of genome size, we used four internal standards to estimate nuclear DNA content in nine insect species with large genomes. The results showed that the combination of two internal standards, *Locusta migratoria* (♂1C = 6.20 pg, ♀1C = 6.60 pg) and *Periplaneta americana*♂ (1C = 3.41 pg), was suitable for estimating large genome of Caelifera by flow cytometry. Using these two internal standards, we estimated the genome sizes of 17 species of Caelifera (12 genera in Acrididae, 2 genera in Pamphagidae, 1 genus in Pyrgomorphidae) using flow cytometry. Genomes ranged from 6.57 pg (*Shirakiacris shirakii*) to 18.64 pg (*Bryodemella holdereri*), the largest described in insects to date. These species showed significant genomic dimorphism based on sex: females had a 0.56 pg larger genome than males on average, which might be due to the sex chromosome determinism mechanism of X0(♂)/XX(♀). To test the results obtained by flow cytometry, we used k-mers of Illumina sequencing data to gauge the C-value of *Calliptamus abbreviatus* and *Haplotropis brunneriana*. The results of the two methods are slightly different. Genomes were estimated to be about 0.28 and 0.26 pg smaller, respectively, than the flow cytometry values. Furthermore, we also reconstructed the evolutionary relationships of these taxa and discuss the genome size evolution in a phylogenetic framework.

## Introduction

Genome size (C-value), or the haploid DNA content of a species, is typically measured in megabases or picograms (1 pg = 978 Mb) (Dolezel et al., 2003). The genome size not only contains genetic information but is also associated with physiological parameters of the organism, such as morphological characteristics of the cells (Gregory, 2001), metabolic rate (Gregory, 2002; Kozłowski et al., 2003), and individual developmental patterns (Griffith et al., 2003).

Genome size has been studied less in invertebrates than in mammals and birds. With more than 1 million insect species described, 1,345 (0.13%) have their genome sizes recorded in the Animal Genome Size Database; this includes only 40 Caelifera species (Gregory, 2020). And recent records indicate about 12,402 Caelifera valid species described on the Orthoptera Species Files (Cigliano et al., 2020). Caelifera exhibit a high degree of variability in C-value, from 3.75 pg for *Warramaba virgo* (Rasch, 1986) to 16.93 pg for *Podisma pedestris* (Westerman et al., 1987), with a mean C-value of 9.34 pg.

Methods of estimating genome size can be divided into two major categories: computational and experimental. The most commonly used experimental methods of estimating genome size in eukaryotes are Feulgen microdensitometry and flow cytometry (Doležel et al., 2007). According to the Animal Genome Size Database, most of the genome sizes of Caelifera were obtained with Feulgen microdensitometry (Gosalvez et al., 1980; Belda et al., 1991). Meanwhile, as the rapid development of next-generation sequencing technology has facilitated research on genomics, k-mer analysis has been used in many insect genome projects because of its feasibility and rationality (Guo et al., 2015; He et al., 2016). Researchers can estimate genome size from sequencing reads by calculating the quotient of the total number of k-mers and the peak frequency distribution.

Flow cytometry is widely used to measure genome size because of its accuracy and speed. Although error from external factors is minimized (Hardie et al., 2002; DeSalle et al., 2005; Hanrahan and Johnston, 2011), the accuracy of estimation relies on internal/external standards (Bennett et al., 2003; Doležel et al., 2007), and there are still challenges selecting appropriate internal standards for different species. The estimated genome size of a given species may vary considerably, depending on the internal standard used (i.e., significant differences in genome size between internal standards and measured species can lead to non-linearity and offset error, resulting in inaccurate measurements) (Bruce Bagwell et al., 1989). Thus, the genome size of an ideal DNA internal standard should be close to that of the target species and not overlap with the peaks produced by the target species. Meanwhile, the internal standard should be easily available, be suitable for flow cytometry protocols, and produce flow histograms with high resolution and reproducibility, as well as accurate genome sizes. These requirements are difficult to satisfy concurrently. To address these problems and to measure nuclear DNA content with a lower coefficient of variance (CV), many researchers use species given by Doležel et al. (1998) for plants and by Tiersch et al. (1989) for animals. Nevertheless, many laboratories have developed their own internal standards. The Animal Genome Size Database^1^ shows 86 internal standards with different nuclear DNA content. Hanrahan and Johnston (2011) estimated the C-values of 134 insects using several internal standards (Gregory, 2020), including the newly proposed *Periplaneta americana* (Hanrahan and Johnston, 2011). He et al. estimated the C-value of nine insects using *Drosophila melanogaster* as an internal standard (He et al., 2016). However, it is difficult to judge the suitability of using plant standards to estimate nuclear DNA content in insects (Gassner et al., 2014).

The recently divergent genome of Acridoidea (Orthoptera, Caelifera) exceeds 6 GB (Dufresne and Jeffery, 2011). Currently, only 5% of insect genomes in the Animal Genome Size Database, most of which belong to Caelifera, are greater than 6 pg (Gregory, 2020). However, there is no suitable internal standard for insects with these large genomes.

In this study, we aimed to determine a suitable internal standard for more accurately measuring large genomes using flow cytometry and to estimate differences in genome size caused by the use of different internal standards. We compared several commonly used internal standards, including *Gallus domesticus*, *Mus musculus*, and *P. americana* and propose here a new internal standard for estimating large insect genomes by flow cytometry: *Locusta migratoria* (♂1C = 6.20 pg), intercontinental distribution in Europe, Asia, and Africa, are an experimental model species with a sequenced genome. Using these internal standards, we used flow cytometry to estimate the genome sizes of 17 species from eight subfamilies in Caelifera. We also performed the k-mer analyses of *Calliptamus abbreviatus* and *Haplotropis brunneriana* to compare and support flow cytometry results. Besides, we used the complete mitochondrial genome to reconstruct the evolutionary history of those 19 species and discuss our results in the light of this phylogenetic hypothesis.

## Materials and Methods

### Species Sampled

Specimens of 17 species of Caelifera were collected from Shaanxi, Ningxia, Inner Mongolia, and Guangxi provinces of China. Information on sample collection is shown in Table 1. For most species, at least three females and three males were used.

**TABLE 1:** List of species showing their collection locality, latitude/longitude, and date in this work.

Subfamily	Species	Collection information		
		Locality	Latitude and longitude	Date
alliptaminae	*Calliptamus barbarus*	Alxa Zuoqi, Alxa League, Inner Mongolia, China	105°51′57′′ E, 38°57′16′′ N	20 July 2019
	*Calliptamus abbreviates*	Changchun, Jilin, China	126°51′21′′ E, 44°52′12′′ N	13 August 2019
		Xi′an, Shaanxi, China	108°52′10′′ E, 34°02′48′′ N	22 August 2019
Eyprepocnemidinae	*Shirakiacris shirakii*	Changchun, Jilin, China	126°51′21′′ E, 44°52′12′′ N	13 August 2019
Melanoplinae	*Pedopodisma tsinlingensis*	Huanggouyu, Weinan, Shaanxi, China	109°34′23′′ E, 34°16′34′′ N	16 August 2019
	*Sinopodisma qinlingensis*	Xunyang Dam, Ankang, Shaanxi, China	108°32′47′′ E, 33°32′51′′ N	5 September 2019
	*Fruhstorferiola huayinensis*	Xunyang Dam, Ankang, Shaanxi, China	108°32′47′′ E, 33°32′51′′ N	5 September 2019
Oedipodinae	*Bryodemella holdereri*	Alxa Zuoqi, Alxa League, Inner Mongolia, China,	105°51′57′′ E, 38°57′16′′ N	20 July 2019
	*Oedaleus asiaticus*	Shizuishan, Ningxia, China	106°21′5′′ E, 39°3′29′′ N	20 July 2019
		Xi’an, Shaanxi, China	108°52′10′′ E, 34°02′48′′ N	22 August 2019
	*Oedaleus infernalis*	Changchun, Jilin, China	126°51′21′′ E, 44°52′12′′ N	13 August 2019
		Xi’an, Shaanxi, China	108°52′10′′ E, 34°02′48′′ N	22 August 2019
	*Epacromius coerulipes*	Ulanhot, Inner Mongolia, China	122°41′42′′ E, 45°43′17′′ N	11 August 2019
		Changchun, Jilin, China	126°51′21′′ E, 44°52′12′′ N	13 August 2019
	*Trilophidia annulata*	Baoji, Shaanxi, China	107°45′29′′ E, 34°19′32″N	28 August 2019
Gomphocerinae	*Pararcyptera microptera meridionalis*	Alxa Zuoqi, Alxa League, Inner Mongolia, China	105°51′57′′E, 38°57′16′′ N	20 July 2019
		Yan’an, Shaanxi	108°52′10′′ E, 34°02′48′′ N	19 June 2019
	*Euchorthippus unicolor*	Xi’an, Shaanxi, China	108°52′10′′ E, 34°02′48′′ N	22 August 2019
Acridinae	*Acrida cinerea*	Xi’an, Shaanxi, China	108°52′10′′ E, 34°02′48′′ N	22 August 2019
Thrinchinae	*Haplotropis brunneriana*	Yan’an, Shaanxi	109°19′39′′ E, 36°54′48′′ N	19 June 2019
	*Filchnerella rubimargina*	Helan Mountain, Yinchuan, Ningxia, China	105°59′17′′ E, 38°43′7′′ N	19 July 2019
Pyrgomorphinae	*Atractomorpha sinensis*	Xi’an, Shaanxi, China	108°52′10″ E, 34°02′48′′ N	22 August 2019

### Sample Preparation and Flow Cytometry

Samples were prepared according to the flow cytometry protocol with slight modification (Brown et al., 2005; Doležel et al., 2007). The heads of the individual insects were used to prepare nuclei, and remaining parts were stored in anhydrous alcohol. Heads of *P. americana*♂ (1C = 3.41 pg) and *L. migratoria*♂ (1C = 6.20 pg), red blood cells of *G. domesticus*♂ (1C = 1.165 pg; which need to be broken by ultrasonic breaker to release the nucleus), and testis tissue of *M. musculus*♂ (1C = 3.30 pg) were used as preparation samples.

Although *G. domesticus* has been widely used as an internal standard (Thindwa et al., 1994; Su et al., 2016), there are considerable differences between strains (Johnston et al., 1999). To avoid inconsistent results due to differences in the genome size of the internal standard, we based the genome size of *G. domesticus* on an average of at least 10 estimates against *P. americana* (1C = 3.41 pg) (Hanrahan and Johnston, 2011). For *L. migratoria*, the 6.60 pg female genome size was estimated with k-mer analysis and flow cytometry (Wang et al., 2014). Male *L. migratoria* was used in this experiment, and the estimated genome size was based on *P. americana*. The estimated internal standards were 1.165 pg (*G. domesticus*) and 6.20 pg (*L. migratoria*), respectively. All estimates had good reproducibility.

Brain tissue from single adult locusts and internal standards was dissected, cut into a Dounce tissue grinder containing 500 μL cold Galbraith buffer (Galbraith et al., 1983), and stroked 35 times with an A pestle. Then 500 μL cold Galbraith buffer was added to clean the pestle, and the solution was filtered through 37 μm nylon mesh into a centrifugal tube to remove cellular debris. Next the solution was centrifuged at 1,000 × *g* for 5 min. The supernatants were discarded, and the precipitates were suspended in 500 μL phosphate-buffered saline (pH 7.2, containing 137 mM NaCl, 2.7 mM KCl, 10 mM Na_2_HPO_4_, 2 mM KH_2_PO_4_). RNase was added to the samples to a final concentration of 20 μg/mL to remove the RNA. Each nucleus solution was subsequently stained with propidium iodide at 4°C for 30 min in the dark until a final concentration of 50 μg/mL. Finally, the sample was filtered once more through a 37 μm nylon mesh filter. Genome size was measured with a NovoCyte flow cytometer with a 488 nm laser. For particle collection, we used an ungated setting and ended collection when the number of nuclei reached more than 20,000 particles.

### Statistical Analyses

Nuclei peaks were obtained with NovoExpress, and the unknown genome size was calculated from the channel numbers of the 2C peaks of each sample as follows:

GS=sampleGS×internalstandard(sample2Cmeanpeakposition/internalstandard2Cmeanpeakposition)

All data analyses were carried out with SPSS Statistics 20. *T*-test or one-way analysis of variance (ANOVA) followed by Tukey multiple-comparisons test were used to compare samples. *P* < 0.05 was considered statistically significant.

### Using K-Mer Analysis to Estimate Genome Size

Hind legs of *C. abbreviatus*♂ and *H. brunneriana*♀ were used to extract DNA by standard methods (Gawel and Jarret, 1991). Experiments, including DNA library preparation and sequencing, were performed according to the standard protocol provided by Illumina. The amount of sequencing data was not less than 300 Gb, which is sufficient for k-mer analysis. DNA libraries with insert sizes of 270 and 500 bp were constructed. An Agilent 2100 Bioanalyzer and quantitative polymerase chain reaction were used to detect fragment sizes and quantify the libraries to determine whether the libraries conformed to the sequencing standards. Each library was sequenced on one lane of a paired end (PE150) with a HiSeq sequencer. Raw reads were handled to slough off low-quality reads (quality score < 20) and duplicate read pairs. To estimate genome size, clean reads were subjected to k-mer distribution by JELLYFISH software (Marçais and Kingsford, 2011), the setting of k-mer size is shown in Supplementary Table S1. And to avoid palindromic sequences and the influence of highly repetitive DNA sequences, with the k-mer size set to 21. Genome size was calculated according to the following formula: genome size = total number of k-mers/peak k-mer frequency distribution (Supplementary Table S2).

To test the content of repeat sequences of k-mer, we used TAREAN, a computational pipeline for identification of repeat from low-pass whole-genome sequence reads (Novak et al., 2017). Clean reads after the above treatment were used to estimate the content of repeat sequence. We randomly selected 2 × 4,800,000 reads and interleave reads in a single file with SeqTK^2^. Then, run TAREAN with default options^3^.

### Mitochondrial Genome Sequencing, Assembly, and Annotation

Information on the DNA-grade tissue samples used in the present study is shown in Table 1. The samples were added to 100% ethanol and stored in a −20°C freezer at the Institute of Zoology of Shaanxi Normal University (Xi’an, Shaanxi, China). Genomic DNA was extracted from the grasshopper leg with a DNeasy Blood and Tissue Kit (QIAGEN Cat. No. 69504) following the manufacturer’s guidelines and stored at −20°C.

Mitochondrial genome sequencing was performed at Biomarker Technologies. The libraries were sequenced on a HiSeq 2500 platform (Illumina) in 150 bp paired-end sequencing mode. Raw sequences were generated in FASTQ format on an Illumina HiSeq sequencing platform. Trimmomatic was used to process reads, including removing adapters and low-quality bases (quality score < Q30). The high-quality sequencing data were *de novo* assembled with Mira 4.0.2 (Chevreux et al., 2004) and MITObim 1.7 (Hahn et al., 2013) with default parameters. Transfer RNA was identified in the MITOS2 Web server^4^ (Bernt et al., 2013). Geneious Prime^5^ (Kearse et al., 2012) was used to compare genes against other related and reference mitogenomes. Results were checked manually to obtain the final mitochondrial genome sequence. The processed file was uploaded to GenBank based on the ORF Finder results.

### Phylogenetic Analyses

To ensure the reliability of the phylogenetic analyses, we included complete mitochondrial genome data, two of which were newly sequenced for this research (*Epacromius coerulipes*: MT499331, *Filchnerella rubimargina*: MK903563.1). The rest of the mtgenomes were obtained from GenBank (Supplementary Table S3). A total of 19 species of insects were used, including 18 ingroup species and 1 outgroup species (*Tetrix japonica*). Phylogenetic analyses were performed on 13 protein-coding genes (PCGs) and 2 rRNA sequences, and multiple alignment was performed on each gene with MAFFT.

Based on the optimization model, phylogenetic analyses used Bayesian inference (BI) using MrBayes version 3.1.2 (Ronquist and Huelsenbeck, 2003), and the maximum likelihood (ML) tree was created with IQTREE 1.7 (Nguyen et al., 2015). The optimization model of BI and ML for nucleotide substitution were the GTR + I + G model and the GTR + F + I + G4 model, respectively, determined by jModelTest (Posada, 2008; Darriba et al., 2012) and ModelFinder (Kalyaanamoorthy et al., 2017). MCMC was run for 1,000,000 generations. The phylogenetic trees were checked and visualized with ITOL version 3 (Letunic and Bork, 2016). The phylogenetic signal of Pagel’s λ and Blomberg’s K in the R package (Caper, Phytools) were used to examine evolutionary patterns in genome size.

## Results

### Comparison of Genome Sizes Measured With Four Internal Standards

**FIGURE 1:**
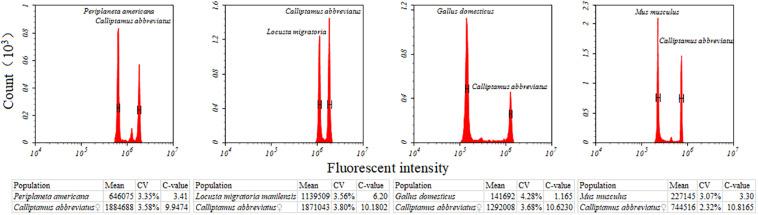
Flow cytometric measurement of the nuclear DNA content of *Calliptamus barbarus*♀ with different internal standards. Four different internal standards were used, including *Gallus domesticus*, *Mus musculus*, *Periplaneta americana*, and *Locusta migratoria*. Estimates concerning the relative DNA staining of nuclei in the copreparation of an insect sample and an internal standard is shown. X-axis = the relative fluorescence intensity of nuclei; Y-axis = number of nuclei.

**FIGURE 2:**
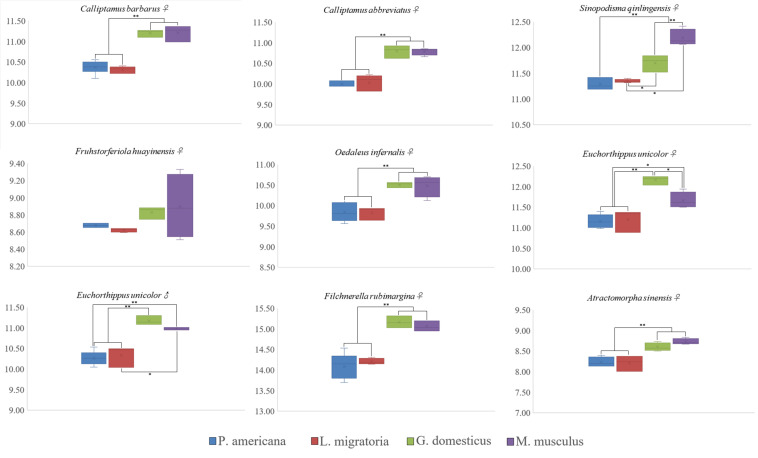
Box and whisker plot showing differences in C-value estimated using four different internal standards. *The mean difference is significant at the 0.05 level. **The mean difference is significant at the 0.01 level. *Gallus domesticus* and *Mus musculus* were used as internal standard to exhibit large C-values (except for *Fruhstorferiola huayinensis*). The C-values estimated by using the *Periplaneta americana* and *Locusta migratoria* as internal standards were similar.

Nine species of Caelifera with a large number of individuals were used to test four different internal standards. A histogram of the peaks obtained with flow cytometry is shown in Figure 1. The estimated C-values differed significantly among the different internal standards (one-way ANOVA and Tukey test; Figure 2 and Supplementary Table S4). The C-values estimated using the testis of *M. musculus* and red blood cells of *G. domesticus* as internal standards were relatively large (except for *Fruhstorferiola huayinensis*). Those estimated using *P. americana* and *L. migratoria* as internal standards did not differ significantly from each other (average difference = 0.0569 pg), but as the C-values of the tested species increased, the difference between the results estimated by the two internal standards also increased.

### Estimating the Genome Sizes of 17 Species of Caelifera With Flow Cytometry

The genome sizes of the 17 species of Caelifera estimated with flow cytometry are shown in Table 2. Except when samples of individual species were insufficient, more than three biological replicates were used in all experiments. The CV of all measured peaks was below 5%. Genome size varied 2.84-fold among the 17 species. The smallest was *Shirakiacris shirakii* at 1C = 6.57 pg (internal standard: *P. americana*). The largest was *Bryodemella holdereri* at 1C = 18.64 pg (internal standard: *L. migratoria*). The genomes of the 17 species of Caelifera exceeded 6 pg (average = 10.80 pg), which indicates that this species has large genomes.

**TABLE 2:** Genome sizes of 17 species estimated by flow cytometry.

Species	Sex	1C value (pg)	Genome size (Mb)	SE (Mb)	n
*Calliptamus barbarus*^*a*^	F	10.31	10, 083	36	5
	M	9.90	9, 679	52	3
*Calliptamus abbreviatus*^*a*^	F	10.03	9, 813	84	5
	M	9.64	9, 424	11	4
*Shirakiacris shirakii*^*a*^	F	7.06	6, 906	75	2
*Shirakiacris shirakii*^*b*^	M	6.57	6, 429	30	4
*Pedopodisma tsinlingensis*^*a*^	F	11.09	10, 847	91	3
*Pedopodisma tsinlingensis*^*b*^	M	10.21	9, 990		1
*Sinopodisma qinlingensis*^*a*^	F	11.35	11, 102	14	5
	M	10.96	10, 719	20	4
*Fruhstorferiola huayinensis*^*a*^	F	8.62	8, 433	10	5
	M	8.30	8, 120	26	4
*Bryodemella holdereri*^*a*^	F	18.64	18, 232	143	3
	M	18.19	17, 787	23	3
*Oedaleus asiaticus*^*b*^	F	9.83	9, 616	59	4
*Oedaleus asiaticus*^*a*^	M	9.24	9, 041	16	4
*Oedaleus infernalis*^*a*^	F	9.83	9, 612	89	3
	M	9.27	9, 070	50	4
*Epacromius coerulipes*^*a*^	F	8.55	8, 362	63	3
	M	8.14	7, 958	20	3
*Trilophidia annulata*^*a*^	F	10.06	9, 840	26	3
	M	9.37	9, 159	46	3
*Pararcyptera microptera meridionalis*^*a*^	F	13.88	13, 579	18	3
	M	13.13	12, 837	37	4
*Euchorthippus unicolor*^*a*^	F	11.20	10, 956	155	3
	M	10.33	10, 107	143	3
*Acrida cinerea*^*a*^	F	11.24	10, 995	48	3
	M	10.64	10, 404	37	3
*Haplotropis brunneriana*^*a*^	F	14.45	14, 130	19	4
	M	13.65	13, 347	10	4
*Filchnerella rubimargina*^*a*^	F	14.21	13, 898	36	4
	M	13.51	13, 211	73	5
*Atractomorpha sinensis*^*a*^	F	8.21	8, 026	104	3
	M	7.55	7, 381	25	3

*1 pg = 978 Mb. F, female; M, male; N, sample size; SE, standard error of estimate. ^a^Locusta migratoria male, 1C = 6.20 pg. ^b^Periplaneta americana, 1C = 3.41 pg.*

### Sex Differences in Genome Size

Genome size was estimated for both males and females of the 17 species. C-values differed significantly by sex (Student *t*-test; Table 3), being larger for females than for males (average difference = 0.56 pg), perhaps because of the sex chromosome determinism mechanism of X0(♂)/XX(♂). The smallest difference (0.32 pg) was in *F. huayinensis*, whereas the largest difference (0.88 pg) was in *Pedopodisma tsinlingensis*.

**TABLE 3:** C-value differences between sex.

Species	Female		Male		*p*
	C-value (pg)	*n*	C-value (pg)	*n*	
*Calliptamus barbarus*	10.31^*a*^	5	9.90^*b*^	3	0.001
*Calliptamus abbreviatus*	10.03^*a*^	5	9.64^*b*^	4	0.009
*Shirakiacris shirakii*	7.06^*a*^	2	6.57^*b*^	4	0.002
*Pedopodisma tsinlingensis*	11.09^*a*^	3	10.21^*b*^	1	0.042
*Sinopodisma qinlingensis*	11.35^*a*^	5	10.96^*b*^	4	0.000
*Fruhstorferiola huayinensis*	8.62^*a*^	5	8.30^*b*^	4	0.000
*Bryodemella holdereri*	18.64^*a*^	3	18.19^*b*^	3	0.030
*Oedaleus asiaticus*	9.83^*a*^	4	9.24^*b*^	4	0.001
*Oedaleus infernalis*	9.83^*a*^	3	9.27^*b*^	4	0.002
*Epacromius coerulipes*	8.55^*a*^	3	8.14^*b*^	3	0.004
*Trilophidia annulata*	10.06^*a*^	3	9.37^*b*^	3	0.000
*Pararcyptera microptera meridionalis*	13.88^*a*^	3	13.13^*b*^	4	0.000
*Euchorthippus unicolor*	11.20^*a*^	3	10.33^*b*^	3	0.016
*Acrida cinerea*	11.24^*a*^	3	10.64^*b*^	3	0.001
*Haplotropis brunneriana*	14.45^*a*^	4	13.65^*b*^	4	0.000
*Filchnerella rubimargina*	14.21^*a*^	4	13.51^*b*^	5	0.000
*Atractomorpha sinensis*	8.21^*a*^	3	7.55^*b*^	3	0.004

*C-values with different letters are significantly different at p < 0.05 (Student t-test). N, number of replicates.*

### K-Mer Analyses of *C. abbreviatus* and *H. brunneriana* Genome Size

**FIGURE 3:**
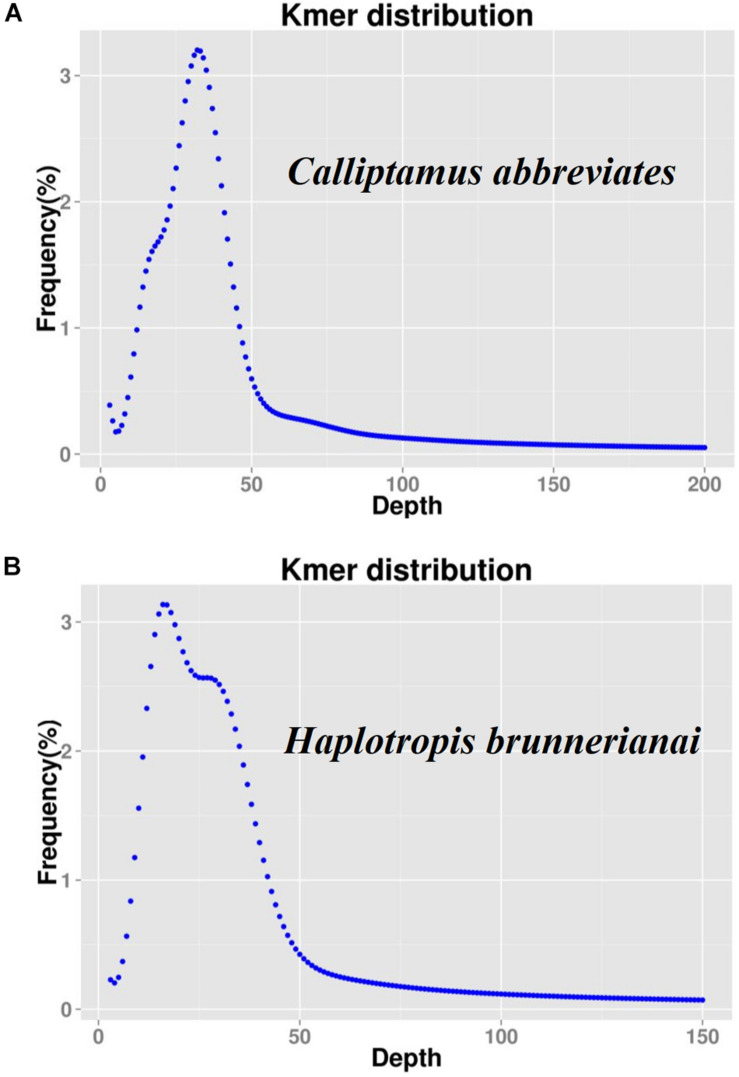
K-mer frequency distribution curve of sequencing reads. The X-axis represents the sequencing depth, and the Y-axis represents the frequency of each k-mer. **(A)** *Calliptamus abbreviates*; **(B)** *Haplotropis brunnerianai*.

K-mer analyses of Illumina sequencing data were used to support the results of *C. abbreviatus*♂ and *H. brunneriana*♀ obtained by flow cytometry (Figure 3). The depth distributions of k-mers showed that the two species had high heterogeneity and a high number of repeat sequences. For *C. abbreviatus*, the C-value obtained with k-mer analysis was 9.36 pg, which was 0.28 pg smaller than that estimated with flow cytometry. According to the distribution of k-mers, the content of repeat sequences was estimated to be about 55.63%, and heterozygosity was about 0.63%. For *H. brunneriana*, the content of repeat sequences and heterozygosity were about 57.58 and 1.40%, respectively, and the C-value based on k-mer analysis was 14.19 pg, which was 0.26 pg smaller than that estimated with flow cytometry. In general, the results of the two methods are slightly different (Table 4). This difference may be due to the fact that analytical methods can considerably influence the values of genome size estimation. In addition, we used TAREAN to further test the content of repeat sequence. The results showed that the content of repeat sequences in *C. abbreviates* and *H. brunneriana* was 51 and 56%, respectively.

**TABLE 4:** C value comparison between flow cytometry and k-mer.

Species	Flow cytometry			K-mer	*p*
	*n*	*SD*	C-value (pg)	C-value (pg)	
*Calliptamus abbreviatus*	4	0.02	9.64	9.36	0.00
*Haplotropis brunneriana*	4	0.04	14.45	14.19	0.01

*C-values with two methods are significantly different at p < 0.05 (one-sample t-test); SD, standard deviation; N, number of replicates.*

### Evolutionary Analyses of Genome Size

**FIGURE 4:**
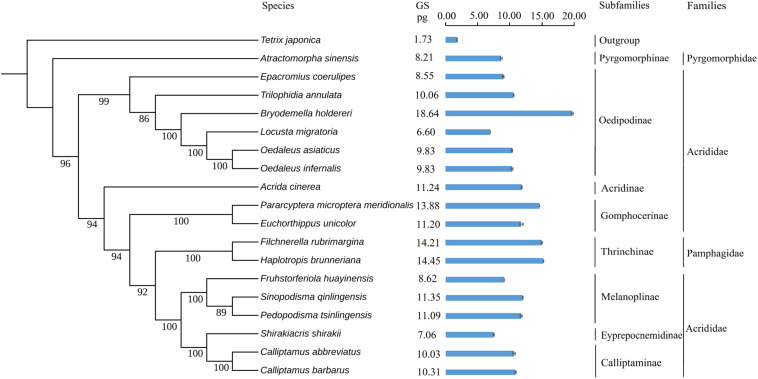
Phylogenetic trees of tested species. Phylogeny introduced the Bayesian inference method (BI) employing the software MrBayes version 3.1.2. The mitochondrial genome sequences of 19 species were retrieved from GenBank. Values indicate posterior probabilities of nodes. *Tetrix japonica* was served as outgroup. The genome sizes (pg) of females are noted on the right for each species. The standard errors (SE) of each species were indicated in the bar chart.

To explore the evolution of genome size in Caelifera, we used ML and BI to reconstruct the present phylogeny in light of mitochondrial genomes containing 18 Caelifera species and 1 outgroup species. The findings supported the morphological classification into subfamilies and families (Figure 4), and the results obtained with the two methods were consistent. The results of these phylogenetic analyses, combined with 13 PCGs and 2 mitochondrial rRNA, basically agreed with previous studies of phylogeny using both mitochondrial and nuclear protein-coding genes (Song et al., 2018).

Tests for the strength and significance of phylogenetic signals of the evolution of genome size using Pagel’s λ and Blomberg’s K showed that the measures of genome size supported a Brownian motion model of evolution and showed complete phylogenetic dependence (λ = 1.00, *K* = 1.18), supporting a neutral evolution mode.

## Discussion

An accurate measurement of genome size is a prerequisite for genome studies (Doležel and Bartoš, 2005), and it also provides data for studying variability in genome size in a large taxonomic group (Gregory et al., 2013). But accurately estimating genome size with flow cytometry requires eliminating potential sources of error (Hardie et al., 2002; DeSalle et al., 2005; Hanrahan and Johnston, 2011). A key factor is the selection of an internal standard (Suda and Leitch, 2010). The present study examined whether different internal standards can significantly influence the estimation of genome size. We performed flow cytometry with four different internal standards to determine the appropriate internal standard for samples with large genomes. Our results make up for errors caused by the inappropriate selection of an internal standard and contributes the research on the Caelifera genome.

The suitability and reproducibility of a set of internal standards were tested, as shown in Supplementary Table S4. The data showed a divergence in the measured nuclear DNA content of Caelifera with larger genomes determined with *G. domesticus* (1C = 1.165 pg), *M. musculus* (1C = 3.30 pg), *P. americana* (1C = 3.41 pg), or *L. migratoria* (1C = 6.20 pg) as internal standards and values derived from flow cytometry.

**FIGURE 5:**
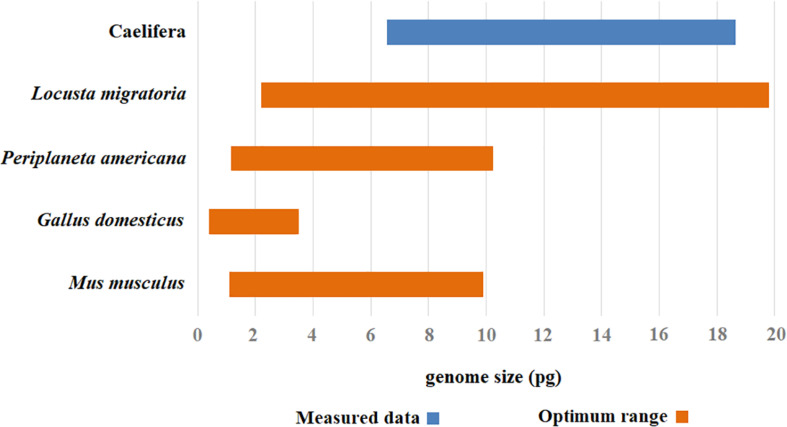
The optimal range of values for different internal standards. The optimal measurement range for each internal standard was assigned color range (the orange), and the range of estimated value in genome size was attributed a corresponding color code (the blue).

Chicken red blood cells have been widely used by some investigators as an internal standard for measuring animal DNA by flow cytometry (Juchno et al., 2010; Jimenez and Kinsey, 2012). A single *G. domesticus* chicken can provide an easy source of cells for many experiments. However, a significant disadvantage of using chicken blood as a standard for flow cytometry is its low level of nuclear DNA compared to many larger insect genomes. Because error increases when the nuclear DNA contents of the standard and the sample differ greatly, the chicken is not a suitable standard for Caelifera with high DNA content. The higher genome size estimated with *M. musculus* as the internal standard may reflect the fact that testis did not grind well with other tissue, resulting in incomplete nuclear release and that actual genome size of *M. musculus* is relatively small. Therefore, *M. musculus* does not provide a true value for flow cytometry estimation of nuclear DNA content. Another potential problem using *M. musculus* as a standard in flow cytometry is its higher cost compared to other internal standards. DNA content estimates for large insect genomes are usually variable, with large standard errors owing to tiny fluctuations in the machine and sample that translate to striking shifts in the standard-to-sample ratio used to measure genome size. In general, the best results were obtained with *L. migratoria* as the internal standard. Within the current measurable range, *L. migratoria* as an internal standard covers the existing genome size of Caelifera. It can be used as an internal standard for insects with genomes ranging from 2 to 20 Gb. Meanwhile, the results also prove that the species works well, producing flow histograms with high resolution and reproducibility. To avoid misinterpretation when the results of query species and the standard are similar in the histograms, we selected *P. americana* as a supplemental internal standard. This species is nearly omnipresent as an urban pest, which makes it easy to collect. To summarize, the combination of two internal standards, *L. migratoria* and *P. americana*, was suitable for measuring the genome size of Caelifera (Figure 5).

We estimated the genome sizes of the remaining Caelifera insects using the set of internal standards determined by the experiments. A total of 17 species (with the exception of the internal standard of *L. migratoria* and the outgroup *T. japonica*) demonstrated a wide range in genome size, from 6.57 to 18.64 pg (Figure 4), with the largest *B. holdereri* at 1C = 18.64 pg (internal standard: *L. migratoria*), which is larger than the recorded largest insect genome of *P. pedestris* (1C = 16.93 pg). The extensive data in the estimated genome size can provide crucial information for subsequent sequencing. However, measurements of genome size reveal only selected information and allow at best a narrow glimpse into current variation in genome size, which do not reflect the complexity of biological and phylogenetic relevance. Further analysis of these data in the context of phylogeny will provide insight into the evolution of the Caelifera genome. These estimates of genome size and the construction of phylogenetic trees showed that the Caelifera genome size is large and varied compared to that of other Insecta suborders. The phylogenetic analyses involved four families (except the outgroup). The nuclear DNA content of Acrididae females ranged from 6.60 pg (*L. migratoria*) (Wang et al., 2014) to 18.64 pg (*B. holdereri*). Only one species of Pyromorphidae was estimated, and the nuclear DNA content of the female was 8.21 pg (*Atractomorpha sinensis*). The genomes of two species of Thrinchinae females ranged from 14.21 pg (*F. rubimargina*) to 14.45 pg (*H. brunneriana*). In addition, the estimated genome sizes of all species in these experiments were greater than 6 pg, and the larger genome of Caelifera may be due to the high number of repeat elements. For example, more than 2,639 repeat families have been identified in the 6.5 Gb genome of *L. migratoria*, accounting for approximately 60% of all genomic components (Wang et al., 2014). We also performed a preliminary estimate of repeat sequence content in *C. abbreviates* and *H. brunneriana*. The results of k-mer and TAREAN analyses yielded slightly different estimates. TAREAN mainly performs graph-based clustering of whole-genome sequence reads with low-pass paired-end, whereas k-mer analysis evaluates repeat sequence content based on k-mer counting algorithm. Differences in the operating principles may affect the results of the analysis. Another reason may be that insufficient coverage paired-end reads were used in TAREAN. But the results of both analyses reported here suggest that as much as 50% of the *C. abbreviates* (1C = 9.36 pg) and *H. brunneriana* (1C = 14.19 pg) genomes might be repeat DNA.

Besides interspecific variation of genome size, a few studies have also revealed intraspecific variation in genome size, including Crustacean arthropod (Jeffery et al., 2016) and insects (e.g., mosquito, Rao and Rai, 1987; Kumar and Rai, 1990; *Tribolium* beetles, Alvarez-Fuster et al., 1991; *Drosophila*, Ellis et al., 2014). In this research, we estimated the genome size of different individuals within a single species; flow cytometry results showed that the maximum and minimum genome size differences of a single species were 0.49 and 0.05 pg, respectively. The genome size variation between individuals of a given species is likely due to the following causes: (i) artifacts of analysis are a primary consideration (Greilhuber, 1998); (ii) another possible explanation is that the intraspecific variation results from the accumulation of genetic differences between geographically isolated populations (Marescalchi et al., 1998; Greilhuber, 2005), and this requires the support of subsequent cytochrome oxidase I sequence analysis; (iii) the influence of unorthodox events (for instance, the different percentages of repetitive DNA caused by differential proliferation/deletion of transposable elements within species). This assumption has been accepted in some cases (Bennetzen et al., 2005) but has not been further studied in Caelifera. Transposable elements, as important components of repeated sequences, have been identified from different Caelifera insects (Bueno et al., 2013; Wang et al., 2014; Camacho et al., 2015), although their direct contribution to intraspecific variation in genome size has not been quantified. In addition, some researchers have described a possible reason for intraspecific variation, not only in the number of repetitive DNA but in sequence complexity as well (Black and Rai, 1988). And our future research should also focus on identifying differences in the type, number, size, and sequence of repeat elements within intraspecific.

Subsequent correlation analyses between genome size and phylogenetic trees were carried out using phylogenetic signals in the R package. To study variation in genome size in the context of phylogeny, special attention must be paid to the measurement of phylogenetic signals. In this study, Pagel’s λ = 1.00 (the evolution of traits followed the evolution of pure Brownian motion models, which rely on phylogeny to explain changes in traits) and Blomberg’s *K* = 1.18 (traits were more similar between relatives than expected). The presence of strong phylogenetic signals (*K* = 1.18, λ = 1.00) suggests that variation in genome size is dependent on phylogenetic patterns.

In addition, similar to the current study, Hanrahan and Johnston (2011) also indicate that marked divergence between sexes cannot be addressed by neglect or merger. They found that five species of insects showed significant sex-based dimorphism in genome size (Hanrahan and Johnston, 2011). Researchers have described a possible cause of differences in genome size based on gender: the sex chromosome determinism mechanism (Liu et al., 2017). Our data showed that females in 17 species of Caelifera exhibited slightly larger genomes than males. Karyotypic analyses reveal that species in the subfamilies Calliptaminae, Eyprepocnemidinae, Melanoplinae, Oedipodinae, Gomphocerinae, Acridinae, Thrinchinae, and Pyrgomorphinae normally exhibit the X0/XX karyotype (Hewitt, 1979), which may explain the difference in genome size between the sexes. Moreover, the analyses of genome size in evolutionary lineages with neo-sex chromosomes (Mesa et al., 1982; Castillo et al., 2010, 2019; Jetybayev et al., 2017) could help to understand several issues about genome size evolution in Caelifera. However, it is too early to explain the subtle divergences in nuclear DNA content between males and females of Caelifera. Further genome size estimation and karyotypic analyses of Caelifera will help to resolve this.

Furthermore, it is worth noting that comparative study of insect genome size shows that Orthoptera is a unique group of Insecta with a significantly enlarged genome (Alfsnes et al., 2017). However, to date, little research has been done on variation in genome size in Orthoptera. This is partly because the high number of repeat sequences hinders to some extent the process of whole-genome sequencing. Thus, the genomes of most animal species that have been sequenced so far (especially invertebrates) are small. In the current research, we estimated the genome sizes of 17 species of Caelifera with an appropriate internal standard for large genomes. Our results can be used to guide whole-genome sequencing and study the important scientific issues associated with variation in genome size.

## Data Availability Statement

The datasets presented in this study can be found in online repositories. The names of the repository/repositories and accession number(s) can be found in the article/Supplementary Material. The data of k-mer analysis can be found from GenBank (PRJNA638780).

## Author Contributions

YH, YM, NZ, YN, and XZ collected specimens. YH contributed to conception and design the experiments and revised the manuscript. YM, NZ, YN, and XZ performed the experiments. YM and NZ analyzed the data. YM wrote the manuscript. All authors contributed to manuscript reading and approved the submitted version.

## Conflict of Interest

The authors declare that the research was conducted in the absence of any commercial or financial relationships that could be construed as a potential conflict of interest.

## Footnotes

1 http://www.genomesize.com

2 https://github.com/lh3/seqtk

3 https://repeatexplorer-elixir.cerit-sc.cz/

4 http://mitos.bioinf.uni-leipzig.de/index.py

5 http://www.geneious.com
