# Supplementary material for: Genome Size of 17 Species From Caelifera (Orthoptera) and Determination of Internal Standards With Very Large Genome Size in Insecta
Source: Front Physiol. 2020 Oct 22;11:567125. doi: 10.3389/fphys.2020.567125 (PMC7642767; doi:10.3389/fphys.2020.567125)
Supplement: Supplementary file 1 [file Table_1.DOCX]

**TABLE S1 | Calculation of the k-mer size**

| Species | c | g (internal standard: Locusta migratoria) | k-mer size |
| --- | --- | --- | --- |
| *Calliptamus abbreviatusa* | 40.64× | 9.64 | 19.27 |
| *Haplotropis brunneria* | 37.10× | 14.45 | 19.50 |

Note. K-mer size = log_4_(c·g); g, genome size; c, sequencing coverage.
