# Supplementary material for: Genome Size of 17 Species From Caelifera (Orthoptera) and Determination of Internal Standards With Very Large Genome Size in Insecta
Source: Front Physiol. 2020 Oct 22;11:567125. doi: 10.3389/fphys.2020.567125 (PMC7642767; doi:10.3389/fphys.2020.567125)
Supplement: Supplementary file 2 [file Table_2.DOCX]

**TABLE S2 | K-mer information for genome size**

| Species | Knum | Kdepth | C-value (Mb) | C-value (pg) |
| --- | --- | --- | --- | --- |
| *C. abbreviatus*♂ | 292,945,995,872 | 32 | 9155 | 9.36 |
| *H. brunneriana*♀ | 416,258,352,360 | 30 | 13875 | 14.19 |

Note. Knum, total number of k-mers; Kdepth, peak k-mer frequency distribution.

Genome size = total number of k-mers/peak k-mer frequency distribution
