# Supplementary material for: Genome Size of 17 Species From Caelifera (Orthoptera) and Determination of Internal Standards With Very Large Genome Size in Insecta
Source: Front Physiol. 2020 Oct 22;11:567125. doi: 10.3389/fphys.2020.567125 (PMC7642767; doi:10.3389/fphys.2020.567125)
Supplement: Supplementary file 3 [file Table_3.DOCX]

**TABLE S3 | Information about mitochondrial genomes19 of species**

| **subfamily** | **Species** | **Accession No.** |
| --- | --- | --- |
| Calliptaminae | *Calliptamus barbarus* | NC_046544.1 |
|  | *Calliptamus abbreviatus* | NC_030626.1 |
| Eyprepocnemidinae | *Shirakiacris shirakii* | NC_021610.1 |
| Melanoplinae | *Pedopodisma tsinlingensis* | NC_032303.1 |
|  | *Sinopodisma qinlingensis* | KX857635 |
|  | *Fruhstorferiola huayinensis* | NC_031379.1 |
| Oedipodinae | *Bryodemella holdereri* | NC_046536.1 |
|  | *Oedaleus asiaticus* | NC_011115.1 |
|  | *Oedaleus infernalis* | NC_029327.1 |
|  | *Epacromius coerulipes* | MT499331 |
|  | *Trilophidia annulata* | NC_027179.1 |
|  | *Locusta migratoria* | NC_014891.1 |
| Gomphocerinae | *Pararcyptera microptera meridionalis* | NC_039962.1 |
|  | *Euchorthippus unicolor* | NC_045237.1 |
| Acridinae | *Acrida cinerea* | NC_014887.1 |
| Thrinchinae | *Haplotropis brunneriana* | MK903563.1 |
|  | *Filchnerella rubimargina* | MT499332 |
| Pyrgomorphinae | *Atractomorpha sinensis* | NC_011824.1 |
| Tetriginae | *Tetrix japonica* | NC_018543.1 |

Note. All sequences, including 13 PCGs and 2mt rRNAs, can be found in GenBank.
