# Supplementary material for: Genome Size of 17 Species From Caelifera (Orthoptera) and Determination of Internal Standards With Very Large Genome Size in Insecta
Source: Front Physiol. 2020 Oct 22;11:567125. doi: 10.3389/fphys.2020.567125 (PMC7642767; doi:10.3389/fphys.2020.567125)
Supplement: Supplementary file 4 [file Table_4.DOC]

**TABLE S4 | Comparison of C-values measured by four internal standards**

| Multiple Comparisons | | | | | |
| --- | --- | --- | --- | --- | --- |
| Dependent Variable | (I) Internal_standard | (J) Internal_standard | Mean Difference (I-J) | Std. Error | p |
| *Calliptamus barbarus* | *P. americana* | *L. migratoria* | 0.06 | 0.07 | 0.829 |
|  |  | *G. domesticus* | -0.83** | 0.09 | 0.000 |
|  |  | *M. musculus* | -0.84** | 0.09 | 0.000 |
|  | *L. migratoria* | *P. americana* | -0.06 | 0.07 | 0.829 |
|  |  | *G. domesticus* | -0.90** | 0.10 | 0.000 |
|  |  | *M. musculus* | -0.90** | 0.10 | 0.000 |
|  | *G. domesticus* | *P. americana* | 0.83** | 0.09 | 0.000 |
|  |  | *L. migratoria* | 0.90** | 0.10 | 0.000 |
|  |  | *M. musculus* | 0.00 | 0.11 | 1.000 |
|  | *M. musculus* | *P. americana* | 0.84** | 0.09 | 0.000 |
|  |  | *L. migratoria* | 0.90** | 0.10 | 0.000 |
|  |  | *G. domesticus* | 0.00 | 0.11 | 1.000 |
| *Calliptamus abbreviatus* | *P. americana* | *L. migratoria* | -0.04 | 0.11 | 0.974 |
|  |  | *G. domesticus* | -0.80** | 0.12 | 0.000 |
|  |  | *M. musculus* | -0.80** | 0.11 | 0.000 |
|  | *L. migratoria* | *P. americana* | 0.04 | 0.11 | 0.974 |
|  |  | *G. domesticus* | -0.76** | 0.11 | 0.000 |
|  |  | *M. musculus* | -0.76** | 0.10 | 0.000 |
|  | *G. domesticus* | *P. americana* | 0.80** | 0.12 | 0.000 |
|  |  | *L. migratoria* | 0.76** | 0.11 | 0.000 |
|  |  | *M. musculus* | 0.00 | 0.11 | 1.000 |
|  | *M. musculus* | *P. americana* | 0.80** | 0.11 | 0.000 |
|  |  | *L. migratoria* | 0.76** | 0.10 | 0.000 |
|  |  | *G. domesticus* | 0.00 | 0.11 | 1.000 |
| *Sinopodisma qinlingensis* | *P. americana* | *L. migratoria* | -0.06 | 0.09 | 0.894 |
|  |  | *G. domesticus* | -0.42** | 0.10 | 0.008 |
|  |  | *M. musculus* | -0.90** | 0.09 | 0.000 |
|  | *L. migratoria* | *P. americana* | 0.06 | 0.09 | 0.894 |
|  |  | *G. domesticus* | -0.35* | 0.09 | 0.011 |
|  |  | *M. musculus* | -0.84** | 0.08 | 0.000 |
|  | *G. domesticus* | *P. americana* | 0.42** | 0.10 | 0.008 |
|  |  | *L. migratoria* | 0.35* | 0.09 | 0.011 |
|  |  | *M. musculus* | -0.48** | 0.09 | 0.002 |
|  | *M. musculus* | *P. americana* | 0.90** | 0.09 | 0.000 |
|  |  | *L. migratoria* | 0.84** | 0.08 | 0.000 |
|  |  | *G. domesticus* | 0.48** | 0.09 | 0.002 |
| *Fruhstorferiola huayinensis* | *P. americana* | *L. migratoria* | 0.06 | 0.15 | 0.981 |
|  |  | *G. domesticus* | -0.15 | 0.17 | 0.791 |
|  |  | *M. musculus* | -0.22 | 0.15 | 0.502 |
|  | *L. migratoria* | *P. americana* | -0.06 | 0.15 | 0.981 |
|  |  | *G. domesticus* | -0.21 | 0.15 | 0.518 |
|  |  | *M. musculus* | -0.28 | 0.14 | 0.229 |
|  | *G. domesticus* | *P. americana* | 0.15 | 0.17 | 0.791 |
|  |  | *L. migratoria* | 0.21 | 0.15 | 0.518 |
|  |  | *M. musculus* | -0.07 | 0.15 | 0.969 |
|  | *M. musculus* | *P. americana* | 0.22 | 0.15 | 0.502 |
|  |  | *L. migratoria* | 0.28 | 0.14 | 0.229 |
|  |  | *G. domesticus* | 0.07 | 0.15 | 0.969 |
| *Oedaleus infernalis* | *P. americana* | *L. migratoria* | 0.01 | 0.15 | 1.000 |
|  |  | *G. domesticus* | -0.67** | 0.15 | 0.004 |
|  |  | *M. musculus* | -0.64** | 0.14 | 0.003 |
|  | *L. migratoria* | *P. americana* | -0.01 | 0.15 | 1.000 |
|  |  | *G. domesticus* | -0.68** | 0.17 | 0.010 |
|  |  | *M. musculus* | -0.67** | 0.16 | 0.008 |
|  | *G. domesticus* | *P. americana* | 0.67** | 0.15 | 0.004 |
|  |  | *L. migratoria* | 0.68** | 0.17 | 0.010 |
|  |  | *M. musculus* | 0.02 | 0.16 | 0.999 |
|  | *M. musculus* | *P. americana* | 0.64** | 0.14 | 0.003 |
|  |  | *L. migratoria* | 0.66** | 0.16 | 0.008 |
|  |  | *G. domesticus* | -0.02 | 0.16 | 0.999 |
| *Euchorthippus unicolor♀* | *P. americana* | *L. migratoria* | -0.04 | 0.14 | 0.990 |
|  |  | *G. domesticus* | -1.00** | 0.14 | 0.000 |
|  |  | *M. musculus* | -0.50* | 0.13 | 0.011 |
|  | *L. migratoria* | *P. americana* | 0.04 | 0.14 | 0.990 |
|  |  | *G. domesticus* | -0.96** | 0.16 | 0.000 |
|  |  | *M. musculus* | -0.46* | 0.15 | 0.038 |
|  | *G. domesticus* | *P. americana* | 1.00** | 0.14 | 0.000 |
|  |  | *L. migratoria* | 0.96** | 0.16 | 0.000 |
|  |  | *M. musculus* | 0.50* | 0.15 | 0.025 |
|  | *M. musculus* | *P. americana* | 0.50* | 0.13 | 0.011 |
|  |  | *L. migratoria* | 0.46* | 0.15 | 0.038 |
|  |  | *G. domesticus* | -0.50* | 0.15 | 0.025 |
| *Euchorthippus unicolor♂* | *P. americana* | *L. migratoria* | -0.07 | 0.13 | 0.950 |
|  |  | *G. domesticus* | -0.91** | 0.13 | 0.000 |
|  |  | *M. musculus* | -0.71** | 0.15 | 0.004 |
|  | *L. migratoria* | *P. americana* | 0.07 | 0.13 | 0.950 |
|  |  | *G. domesticus* | -0.84** | 0.14 | 0.001 |
|  |  | *M. musculus* | -0.65* | 0.16 | 0.013 |
|  | *G. domesticus* | *P. americana* | 0.91** | 0.13 | 0.000 |
|  |  | *L. migratoria* | 0.84** | 0.14 | 0.001 |
|  |  | *M. musculus* | 0.20 | 0.16 | 0.634 |
|  | *M. musculus* | *P. americana* | 0.71** | 0.15 | 0.004 |
|  |  | *L. migratoria* | -0.65* | 0.16 | 0.013 |
|  |  | *G. domesticus* | -0.20 | 0.16 | 0.634 |
| *Filchnerella rubimargina* | *P. americana* | *L. migratoria* | -0.13 | 0.14 | 0.807 |
|  |  | *G. domesticus* | -1.08** | 0.15 | 0.000 |
|  |  | *M. musculus* | -0.98** | 0.15 | 0.000 |
|  | *L. migratoria* | *P. americana* | 0.13 | 0.14 | 0.807 |
|  |  | *G. domesticus* | -0.95** | 0.16 | 0.000 |
|  |  | *M. musculus* | -0.85** | 0.16 | 0.001 |
|  | *G. domesticus* | *P. americana* | 1.08** | 0.15 | 0.000 |
|  |  | *L. migratoria* | 0.95** | 0.16 | 0.000 |
|  |  | *M. musculus* | 0.10 | 0.17 | 0.926 |
|  | *M. musculus* | *P. americana* | 0.98** | 0.15 | 0.000 |
|  |  | *L. migratoria* | 0.85** | 0.16 | 0.001 |
|  |  | *G. domesticus* | -0.10 | 0.17 | 0.926 |
| *Atractomorpha sinensis* | *P. americana* | *L. migratoria* | 0.03 | 0.08 | 0.988 |
|  |  | *G. domesticus* | -0.37** | 0.07 | 0.001 |
|  |  | *M. musculus* | -0.50** | 0.08 | 0.000 |
|  | *L. migratoria* | *P. americana* | -0.03 | 0.08 | 0.988 |
|  |  | *G. domesticus* | -0.40** | 0.09 | 0.002 |
|  |  | *M. musculus* | -0.53** | 0.09 | 0.000 |
|  | *G. domesticus* | *P. americana* | 0.37** | 0.07 | 0.001 |
|  |  | *L. migratoria* | 0.40** | 0.09 | 0.002 |
|  |  | *M. musculus* | -0.13 | 0.08 | 0.399 |
|  | *M. musculus* | *P. americana* | 0.50** | 0.08 | 0.000 |
|  |  | *L. migratoria* | 0.53** | 0.09 | 0.000 |
|  |  | *G. domesticus* | 0.13 | 0.08 | 0.399 |

Note. * The mean difference is significant at the 0.05 level, ** The mean difference is significant at the 0.01 level. (One-way ANOVA, followed by Tukey’s test); *Periplaneta americana*, 1C=3.41pg; *Locusta migratoria*, 1C=6.20pg; *Gallus domesticus*, 1C=1.165pg; *Mus musculus*, 1C=3.30pg
